# Improved Manufacturability and In Vivo Comparative Pharmacokinetics of Dapagliflozin Cocrystals in Beagle Dogs and Human Volunteers

**DOI:** 10.3390/pharmaceutics13010070

**Published:** 2021-01-07

**Authors:** Sangho Cho, Jeongwook Lee, Yongwon Yoo, Minyong Cho, Seil Sohn, Beom-Jin Lee

**Affiliations:** 1 Daewon Pharm. Co., Ltd., 520 Cheonhodae-ro, Gwangjin-gu, Seoul 04994, Korea; shcho@daewonpharm.com (S.C.); ljw5081@daewonpharm.com (J.L.); yongwon@daewonpharm.com (Y.Y.); cmy777@daewonpharm.com (M.C.); sisohn1@daewonpharm.com (S.S.); 2 College of Pharmacy, Ajou University, Suwon 16499, Korea

**Keywords:** dapagliflozin, cocrystal, dry granulation, manufacturability, stability, dissolution, comparative pharmacokinetics

## Abstract

Dapagliflozin (DAP), which improves glycemic control in patients with type 2 diabetes mellitus, has poor physical properties against heat and moisture, thus hindering its manufacturing potential. The superior physicochemical properties of a recently developed cocrystal of DAP and citric acid (DAP cocrystal) in comparison with those of DAP and Forxiga^®^, a patented solvate form with propandiol monohydrate, were identified via structural analysis and moisture sorption isotherm. For the first time, the formulation, manufacturability, and in vivo bioavailability of DAP cocrystals were successfully investigated to develop oral dosage forms that substitute Forxiga^®^. The intrinsic dissolution rate of DAP cocrystal was controlled by varying particle size distribution. Unlike the direct compression (DC), roller compaction (RC) was more preferable to obtain good flowability of dry granules for a continuous manufacturing system. The cocrystal structure was maintained throughout the stability assessment period. In Vitro dissolution pattern differences of the optimized DAP cocrystal tablet with RC and the reference tablet, Forxiga^®^ 10 mg, were pharmaceutically equivalent within 5% in four different media. Furthermore, comparative pharmacokinetic analysis confirmed that a 10 mg DAP cocrystal tablet with RC was bioequivalent to a 10 mg Forxiga^®^ tablet, as assessed in beagle dogs and human volunteers.

## 1. Introduction

Sodium glucose transport proteins (SGLTs), transporting sodium and glucose, are known to have six subtypes (SGLT1-6) [1]. In particular, SGLT2 is involved in re-absorbing glucose from the glomerular filtrate into the systemic circulation. In healthy individuals, all filtered glucose is reabsorbed into the proximal renal tubule by SGLT2 and then returned to the bloodstream [2,3]. Dapagliflozin (DAP), is a recently developed SGLT2 inhibitor that inhibits glucose reabsorption from the renal tubules and prevents it from returning into the bloodstream. In addition, DAP has a weight loss effect by removing calories through glucose excretion, thus lowering the risk of type 2 diabetes mellitus complications [4]. Most of the existing hypoglycemic drugs increase insulin (the blood glucose regulating hormone) sensitivity or secretion. DAP has an insulin-independent mechanism that lowers glycated hemoglobin and does not stimulate pancreatic beta cells [5].

However, DAP has poor solid stability and a difficult formulation process owing to its high hygroscopicity [6]. To compensate for these properties, immediate release tablets consisting of DAP with propanediol and water (dapagliflozin propanediol monohydrate, DAP-PH) were created and commercialized. However, these tablets have poor thermal stability due to the low melting point (about 70 °C) of DAP-PH, and they easily return to an amorphous form, owing to the intrinsic amorphous nature of DAP [7]. Therefore, appropriate management of temperature and humidity is required to successfully manufacture DAP tablets.

Substantial efforts are being expended to develop a new crystalline form or multi-component crystal (cocrystal, co-amorphous, salt, solvate/hydrate) to improve the physical properties of the active pharmaceutical ingredient (API) [8,9]. Among the multi-component crystals, cocrystals have recently emerged as a new method for improving the properties of various APIs [10]. Cocrystals are based on hydrogen bonding interactions between the API and a coformer, homo (hydrogen bonds between homo-functional groups) and hetero synthons (hydrogen bonds between heterogeneous functional groups) in the same crystalline grid [11,12]. Cocrystal technology has the advantage that a covalent bond to the API does not need to be created or broken [13]. In addition, new cocrystallization can be performed for all types, such as ionized or non-ionized API [14]. Our group selected a Generally Recognized as Safe (GRAS) list of 63 coformers that might form cocrystals with DAP while avoiding existing DAP-PH patents (Table S1). X-ray diffraction analysis of the five candidates showed new crystalline signals, namely citric acid (CA), L-proline, sodium lauryl sulfate, L-tryptophan, and zinc chloride (Figure S1). Among them, CA was chosen after excluding other coformers in which the mixture signal appeared and the phase transition was detected at 60 °C. Although the cocrystal of DAP and CA (DAP cocrystal) was synthesized to investigate its physical properties, the detailed experimental approaches regarding formulation, manufacturing process, and pharmacokinetic (PK) comparison were not studied [7].

The aim of this work was to compare DAP cocrystals with amorphous DAP and DAP-PH. The physical properties against heat and moisture, and DAP cocrystal-based granulation for tableting with good flowability and continuous manufacturing flow, RC process to optimize process parameters such as roller speed, screw feeder speed, and roll pressure. Then, In Vitro dissolution and PK profiles of the optimal cocrystal tablet and commercialized DAP tablet, Forxiga^®^ 10 mg (AstraZeneca Co., Lts., Seoul, Korea) as a reference tablet, were compared in beagle dogs. Finally, comparative pharmacokinetics in healthy human volunteers were also investigated to ensure bioequivalence between the DAP cocrystal tablet and Forxiga^®^ 10 mg.

## 2. Materials and Methods

### 2.1. Materials

DAP-CA cocrystal (99.9% purity) was obtained from Sun Pharm. Industries, Ltd. (Mumbai, India). Anhydrous lactose (Supertab 21AN) was obtained from DFE Pharma (Goch, Germany). Lactose monohydrate (Tablettose) was obtained from Meggle Pharma (Wasserburg, Germany). Microcrystalline cellulose (Avicel PH-101) was purchased from FMC biopolymer (Philadelphia, PA, USA). Microcrystalline cellulose (Vivapur 12) was obtained from JRS Pharma (Rosenberg, Germany). Mannitol (Pearlitol 100 SD) was obtained from ROQUETTE Pharma (Lestrem, France), crospovidone (Polyplasdone XL) was provided by Ashland (DA, USA), colloidal silicon dioxide (Aerosil 200 pharma) was purchased from Evonik (Essen, Germany), and magnesium stearate was purchased from FACI (Genova, Italy). Opadry was purchased from Colorcon (Stoughton, WI, USA). DAP amorphous (99.9% purity) and DAP-PH (99.9% purity) were provided by Dr. Reddy (Hyderabad, India), and Forxiga^®^ 10 mg, a control agent, was provided by AstraZeneca Korea (Seoul, Korea).

### 2.2. DAP Cocrystal Characterization

#### 2.2.1. X-ray Diffraction (XRD)

XRD was performed to confirm the DAP cocrystal structure. DAP, CA, DAP cocrystal, and a physical mixture of DAP and CA (1:1 ratio) samples were prepared for the analysis. It was performed on a DMAX2500/PC X-ray diffractometer (Rigaku, Tokyo, Japan) equipped with a scintillator counter using Cu Kα radiation (λ = 1.5418 Å), generated at 45 kV and 40 mA. First, the sample was placed in the holder and pressed down to obtain a smooth and flat surface. The analysis angle was in the range of 3–40° (2θ) with a step size of 0.02° (2θ).

#### 2.2.2. Fourier Transform Infrared Spectroscopy (FTIR)

FTIR was performed to investigate the molecular motion and molecular structure of the DAP cocrystal to confirm its formation. DAP, DAP-PH, DAP cocrystals, and CA were prepared. FTIR analysis was performed on a Smart iTX infrared spectrometer (Thermo Fisher, Waltham, MA, USA). A total of 64 scans were collected with a resolution of 0.2 cm^−1^ for each sample in the range of 4000 to 400 cm^−1^.

#### 2.2.3. Differential Scanning Calorimetry (DSC)

DSC was performed to compare the thermal stabilities of DAP, DAP-PH, and DAP cocrystals. DSC measurements were performed on a Universal V4.5A instruments (TA Instrument, New Castle, DE, USA) instrument. The prepared samples were placed in a closed aluminum pan and heated at a rate of 10 °C/min under nitrogen purging. The sample scan started at 30 °C and ended at 300 °C.

#### 2.2.4. Thermogravimetric Analysis (TGA)

TGA was performed using a TGA 2 star system (Mettler Toledo, Greifensee, Switzerland) instrument. The sample was placed in an aluminum pan and heated at a constant rate of 10 °C/min under nitrogen purging. The sample scan started at 30 °C and ended at 500 °C.

#### 2.2.5. Dynamic Vapor Sorption (DVS)

DVS experiments were conducted using a VTI-SA+ instrument (TA Instruments, USA). All samples were dried for 1 h under a stream of nitrogen to reach the equilibrium dry mass. The relative humidity (RH) sequence was varied from 0% to 95% RH with 10% RH increments. The temperature remained constant at 25 ± 0.1 °C. The sorption/desorption isotherms were calculated from the equilibrium mass values.

### 2.3. Solubility Test

The solubility of DAP, DAP-PH, and DAP cocrystals in equilibrium was analyzed in four media (pH 1.2, 4.0, 6.8, distilled water (pH = 6.5) under various conditions. Each medium was prepared according to Table S2. Excess DAP, DAP-PH, and DAP cocrystal powder were added to the set medium and shaken at 25 °C for 24 h. The sample was separated at 12,000 rpm for 5 min, centrifuged to collect the supernatant, diluted, and analyzed by high performance liquid chromatography (HPLC). To evaluate solubility, the dose number (D_0_) was calculated according to Equation (1):

D_0_ = (M_0_/V_0_)/C_S_ (1)

M_0_ is the maximum dose per administration (mg), C_S_ is the solubility (mg/mL), and V_0_ is 250 mL. The largest administered DAP dose (M_0_) was 10 mg.

### 2.4. Appearance and Stability Test of API

After storing DAP, DAP-PH, and DAP cocrystals under different temperature and humidity conditions, changes in appearance were observed. The three storage conditions were: 60 °C in a closed vial, 90% relative humidity (RH) with an open vial at 25 °C, and 75% open vial at 40 °C. The first storage test represents applied heat and moisture, both separately and to each other. The samples were stored for 1 week on Petri dishes to identify appearance changes. Stability tests were performed under the above-mentioned storage conditions. Drug content and impurities of DAP, DAP-PH, and DAP cocrystals after storage for 4 weeks were determined by a HPLC assay as described below.

### 2.5. Scanning Electron Microscopy (SEM)

The DAP cocrystal morphology was examined using an EM 30 (COXEM, Daejeon, Korea) scanning electron microscope at a voltage of 20 kV. The samples were mounted on the brass stage using carbon tape and coated with gold for 250 s under an argon atmosphere by using an automatic sputter coater.

### 2.6. Manufacturing DAP Cocrystal Tablets by Direct Compression

#### 2.6.1. DAP Cocrystal Tablets Preparation

DAP cocrystal-containing tablets were first prepared using a DC method. DAP cocrystal, microcrystalline cellulose (Vivapur 12), and colloidal silicon dioxide (Aerosil 200 pharma) were mixed using a V-mixer and passed through a 16-mesh sieve. The mixtures were blended with mannitol (Pearlitol 100 SD), lactose monohydrate (Tablettose 80), and crospovidone (Polyplasdone XL) using a V-mixer and passed through a 16-mesh sieve. The granules were blended with magnesium stearate using a V-mixer and then compressed using a rotary tablet press machine (Piccola Euro B-10 type; Riva, Buenos Aires, Argentina) using diamond-shaped punches (I Holland, Long Eaton, UK). Table 1 shows detailed formulation compositions (mg) of the DAP cocrystal-loaded tablet via DC process.

#### 2.6.2. DAP Cocrystal Milling Effect

The DAP cocrystal particle size reduction was done in a jet mill (Jet-O-Mizer^TM^, Telford, PA, USA) with a grinding pressure of approximately 8 bars. The size distribution of the milled DAP cocrystal was measured with a HELOS particle size analyzer (Sympatec GmbH, Clausthal-Zellerfeld, Germany) with 1 bar disperse pressure. The particle size reduction effect was assessed by an In Vitro dissolution test. The dissolution rate was evaluated using a dissolution tester (Hanson Research Co., Los Angeles, CA, USA) according to dissolution test method 1 of USP 41. The dissolution test medium was a pH 1.2 aqueous solution. The stirring speed of the paddle was 50 rpm and the volume of the elution medium was 900 mL. The media temperature was maintained at 37 ± 0.5 °C during the 2 h evaluation time. The dissolution rate of the control, Forxiga^®^ 10 mg, was also evaluated under the same conditions.

### 2.7. Manufacturing DAP Cocrystal Tablets by Roller Compaction

#### 2.7.1. Preparation Process of DAP Cocrystal Tablets

DAP cocrystal-containing tablets were prepared using a dry granulation method. Briefly, DAP cocrystal, microcrystalline cellulose (Avicel pH 101), and crospovidone (Polyplasdone XL) were mixed using a V-mixer and passed through a 16-mesh sieve. The mixtures were blended with microcrystalline cellulose (Avicel pH 101), lactose anhydrous (Supertab 21AN), and colloidal silicon dioxide (Aerosil 200 pharma) using a V-mixer and passed through a 16-mesh sieve. The mixtures were blended with magnesium stearate using a V-mixer and granulated using a TF-mini roller compactor (Freund Corp., Tokyo, Japan) instrument. The dry granulated sheets were passed through a 16-mesh sieve. The granules were blended with colloidal silicon dioxide (Aerosil 200 pharma) and magnesium stearate using a V-mixer, and then compressed using a rotary tablet press machine using diamond-shaped punches. Table 2 gives detailed formulation compositions (mg) of the DAP cocrys-tal-loaded tablet via RC process.

#### 2.7.2. RC Manufacturing Optimization

To check the effect of the roller and screw feeder rotation speeds on granule properties, granules were prepared with roll rotation speeds of 2, 5, and 8 rpm and screw feeder rotation speeds of 1, 4, 7, and 10 rpm. After achieving synchronized feeding speed, the roller pressure effect was evaluated with roller pressures of 1.5, 3.5, and 6.0 MPa. The roller pressure influence was evaluated using a dissolution test with an aqueous solution medium (pH 1.2).

#### 2.7.3. Effect of Tablet Hardness on DAP Cocrystal Release

Owing to the manufacturing characteristics of the RC granulation method, the produced pressure-sensitive granules have a decisive influence on the tablet properties as they are additionally pressed once more in the tableting process. Therefore, during this process, tablets were prepared according to the pressure applied to evaluate their dissolution profiles. The maximum tablet hardness was measured at 13.0 kp. Thus, tablet hardness values of 1, 4, 7, 10, and 13 were selected for analysis. The dissolution test medium was a pH 1.2 aqueous solution.

#### 2.7.4. Amount of Disintegrant

The dissolution rate of the DAP cocrystal was evaluated according to the amount of disintegrant, Polyplasdone XL, to obtain a dissolution profile similar to Forxiga^®^ 10 mg. The amount of Polyplasdone XL was formulated with 5, 7.5, and 10 mg per each tablet. The dissolution medium was pH 1.2 and pH 4.0 aqueous water solution. The dissolution rate of the control, Forxiga^®^ 10 mg, was also evaluated under the same conditions.

#### 2.7.5. In Vitro Dissolution Test of DAP Cocrystal Tablet and Forxiga^®^ 10 mg

The dissolution rate of the optimized DAP cocrystal tablet was compared with that of Forxiga^®^ 10 mg. The dissolution medium had pH of 1.2, 4.0, or 6.8, or was an aqueous water solution. The stirring speed was 50 rpm, the capacity of the elution medium was 900 mL, and the temperature was tested at 37 ± 0.5 °C. The dissolution rate was evaluated for 2 h at pH 1.2, 4.0, and 6.8 and for 6 h in water. Each dissolution test was terminated when the set time was reached, or the DAP dissolution was more than 90%.

### 2.8. Stability Assessment

#### 2.8.1. Stability Assessment of DAP Cocrystal Tablets

According to the ICH guidelines, stability tests were performed under accelerated and long-term storage conditions. DAP cocrystal tablets were packed into a Press Trough Package blister pack (alu-alu foil) and served as the stability test samples. For accelerated stability, samples were stored at 40 °C and 75% RH over 6 months. For long-term storage stability, samples were stored at 25 °C and 60% RH over 12 months. Drug content and impurities were determined by HPLC as described below.

#### 2.8.2. Stability Assessment of Cocrystal Structure Maintenance

XRD was performed to confirm whether the DAP cocrystal structure was maintained during the stability test. The DAP amorphous, CA, and DAP cocrystals were prepared for comparison with tablet samples. Tablets consisting of DAP, DAP cocrystal, and a mixture of DAP and CA (1:1) were prepared. In addition, among the excipients used, lactose anhydrous with a crystal structure and a placebo excluding API were also prepared. The analysis was conducted after a 6-month storage under long-term and accelerated storage conditions.

### 2.9. HPLC Determination of DAP Content and Impurities

DAP content and impurities were analyzed using HPLC. For the drug content test, 20 tablets were weighed individually, crushed into a fine powder, and mixed together to give a sample containing 50 mg DAP. The diluent solution, a mixture of distilled water and acetonitrile (1:1), was added to a volumetric flask and the drug was extracted for 2 min at 60 Hz using a bath sonicator (8510-DTH; Branson, Danbury, CT, USA). The final DAP concentration was determined to be 50.0 μg/mL. DAP concentration was determined using an Agilent 1260 HPLC system (Agilent Technologies Inc., Santa Clara, CA, USA). Chromatographic separation was performed using a Kromacil^®^ C18 column (5 µm, 4.6 mm × 150 mm) at a flow rate of 1.0 mL/min with a mobile phase consisting of 10 mM potassium dihydrogen phosphate (pH was adjusted to 2.00 with phosphoric acid) and acetonitrile at a 55:45 ratio (*v*/*v*). The sample was injected, and the peaks were monitored using a UV detector at 225 nm. The dissolution test sample was analyzed under the same conditions as the content test.

To assess impurities, the HPLC system and Kinetex XB-C18 column (2.6 µm, 4.6 mm × 100 mm, Phenomenex^®^, Torrence, CA, USA) were used to perform chromatographic separation at a flow rate of 1.0 mL/min with a gradient elution, done by changing the ratio of mobile phase A to mobile phase B. Mobile phase A consisted of a pH 5.0 buffer solution [about 1.36 g of ammonium dihydrogen phosphate dissolved in 1000 mL water, then completely dissolved by ultrasonic shaking, adjusted to pH 5.0 with a mixture of ammonia and water (1:9), and filtered with a 0.22 μm filter] and acetonitrile at a 9:1 ratio (*v*/*v*). Mobile phase B consisted of pH 5.0 buffer solution and acetonitrile at a 2:8 ratio (*v*/*v*).

The standard calibration curve was constructed in the range of 0.0–13.19 μg/mL DAP concentration and three independent measurements were performed at each concentration. Linear regression analysis was used for the linear relationship between peak area and DAP concentration, obtaining the regression equation y = 27.588x − 3.3068 and a regression coefficient of 0.9997. The accuracy study was in triplicate performed for 80%, 100% and 120% of DAP after injecting into HPLC system. The recovery rate of DAP ranged from 98.0% to 102%, and the relative standard deviation was less than 1%. The precision of method was analyzed by repeatability of sample injection. Peak areas and retention times were obtained by determining six replicates of fixed amount of drug. The relative standard deviation of DAP was less than 0.1%. The impurity verification method was conducted according to the API manufacturer’s method.

### 2.10. PK Study in Beagle Dogs

The PK test in beagle dogs was performed at the Chemon Non-Clinical Research Institute (Gyeonggi-do, Korea) and approved by the Ethics Committee (Protocol number: 18-D304). Twelve beagle dogs were kept on an empty stomach overnight, followed by administration of a DAP cocrystal tablet and Forxiga^®^ 10 mg to each of the six beagle dogs. After the first dose, the drug was washed out for 7 days, and the second phase was cross-dose. Water intake was discontinued 2 h before drug administration, and all beagle dogs were supplied with water and food 4 h after the first dose. Blood samples were collected at predetermined time points (0.25, 0.50, 0.75, 1, 1.5, 2, 3, 4, 6, 8, and 24 h). All blood samples were centrifuged at 3000× *g* for 10 min, and aliquots of plasma were stored at −80 °C until analyzed using the validated LC-MS/MS method. Plasma concentration of DAP was determined by LC–MS/MS (Agilent 1260; Agilent Technologies Inc., Santa Clara, CA, USA). Chromatographic separation of DAP was performed using an ODS column (Capcellpak C18 ACR, 4.6 × 150 mm, 5 mm, Shiseido, Tokyo, Japan)

### 2.11. PK Study in Human Volunteers

Human PK studies were approved by the Ethics Committee (Protocol no.: DW2701_P102, Chungbuk National University Hospital, Cheong-Ju, Korea) under a randomized, open-label, 2-way crossover design. In total, 37 subjects (age, 23.3 ± 3.4 years; weight, 72.8 ± 10.8 kg; height, 174.3 ± 5.1 cm; BMI, 23.9 ± 2.8 kg/m^2^) participated in the study after providing written informed consent. Participants were fasted except for drinking water to maintain an empty stomach for at least 10 h. Around 8 am, subjects in each group orally administered one DAP cocrystal tablet and one Forxiga^®^ 10 mg tablet with 150 mL of water in the order of subject number. During the dosing time, oral examination was performed on each volunteer to prevent chewing or splitting of the tablet. After the first dose, the drug was washed out for 7 days, and the second phase was cross-dose. Blood samples were collected at predetermined time points (0, 0.167, 0.33, 0.5, 0.75, 1, 1.5, 2, 3, 4, 6, 8, 12, 16, 24, 36, and 48 h). All blood samples were centrifuged at 3000× *g* for 10 min, and aliquots of plasma were stored at −80 °C until analyzed using the validated LC-MS/MS method. Plasma concentration of DAP in human plasma samples was also determined by LC–MS/MS according to the same procedures in beagle dogs

The detailed dosages and dosing times for PK studies were performed based on the clinical data of Forxiga^®^ 10 mg tablet, reporting that the T_max_ of DAP and T_1/2_ are between 1 and 2 h and 10–12 h, respectively after oral administration [4].

### 2.12. PK Analysis

Non-compartmental PK statistical analysis was performed using Phoenix WinNonLin (version 8.1, Pharsight, Sunnyvale, CA, USA). The maximum plasma concentration (C_max_) and time to reach it (T_max_) after oral administration were directly determined from the plasma concentration-time curve. The area under the plasma concentration-time curve (AUC) was computed using the linear trapezoidal rule as follows:

AUC_t1-t2_ = (t_2_ − t_1_) × (C_1_ + C_2_)/2

where AUC_t1−t2_ is the area under the plasma concentration-time curve during time t_1_ to time t_2_, and C_1_ and C_2_ are the plasma concentrations at times t_1_ and t_2_, respectively.

## 3. Results

### 3.1. DAP Cocrystal Characterization

Prior to manufacturing tablets, characterization was performed to evaluate the properties of DAP cocrystal and to reconfirm its enhanced thermal and water resistance properties identified in previous reports. The cocrystal structure formation was confirmed by XRD, as shown in Figure S2A. The DAP cocrystal showed peaks at 2θ values of 4.48°, 8.84°, and 12.28°, which did not appear in DAP and CA. In particular, the peak at 4.48° is considered crucial for confirming the presence or absence of crystals. The formation of hydrogen bonds in the cocrystal structure was confirmed by the FTIR spectra, as shown in Figure S2B. The sharp-shaped O–H bond in the DAP cocrystal was confirmed at 3546, 3465, and 3401 cm^−1^, which was not observed for DAP and DAP-PH. This intense O–H bond signal was derived from the CA absorption peak shift at 3493 and 3275 cm^−1^. In addition, the C=O bond of the carboxylic acid in the DAP cocrystal was identified at 1716 and 1691 cm^−1^, showing a red shift of the CA absorption peak at 1742 and 1693 cm^−1^ [15]. The thermal and water resistance properties were characterized by DSC, TGA, and dynamic vapor sorption. From Figure S2C, enhancement of the thermal properties was identified at the DAP cocrystal melting point of approximately 90 °C, which was higher than that of DAP and DAP-PH. The decomposition phenomenon was well matched with the melting point of the DAP cocrystal. The initial DAP cocrystal weight loss was identified above 160 °C, showing a higher thermal stability than DAP and DAP-PH (Figure S2D). The dynamic vapor sorption isotherms were measured to compare their stability against humidity. As shown in Figure S3, the DAP cocrystal showed the strongest moisture resistance with low water uptake at all humidity conditions.

### 3.2. Solubility Test

DAP is known as a nonionic drug with a pKa of 12.6, which does not cause a significant difference in solubility according to pH. Table 3 shows that DAP-PH and the DAP cocrystal, which have a multi-component crystal structure compared to the amorphous DAP, have improved solubility compared to the amorphous form. The dose number (D_0_) of all drugs was less than 1.0, indicating a high solubility group. D_0_ represents the ratio of the drug concentration in the medium (250 mL) administered in the PK clinical trial and the saturated solubility in the medium. According to FDA guidelines, when the drug dose (mg)/solubility (mg/mL) is ≤250 mL, the drug is considered to have high solubility, equal to a D_0_ value < 1.0.

### 3.3. Appearance and Stability Test of Active Pharmaceutical Ingredient (API)

An appearance test was conducted to determine how the storage conditions affect the properties of APIs. Figure S4 shows the appearance changes of DAP, DAP-PH, and DAP cocrystal when exposed to either heat, moisture, or both. DAP changed to a liquid phase under all storage conditions. In addition, the DAP-PH phase was observed at 60 °C due to its low melting point, as shown in the DSC results. The heat and moisture effects were also identified in the DAP-PH sample, which resulted in sticky and hardened powder. In contrast, the phase transition of the DAP cocrystal was not observed in all storage conditions, which means it remained in a solid state. These results are well correlated with the characterization results mentioned above. Accelerated stability under heat and moisture conditions was evaluated by content and impurity analysis of APIs. Table S3 shows that DAP content and impurities did not change in the three APIs despite the phase transition phenomenon, especially in DAP and DAP-PH. This result implied that DAP stability was not influenced by intrinsic stability but was affected by combination with the excipient when phase transition of DAP had occurred.

### 3.4. Manufacturing DAP Cocrystal Tablets by Direct Compression

**Figure 1:**
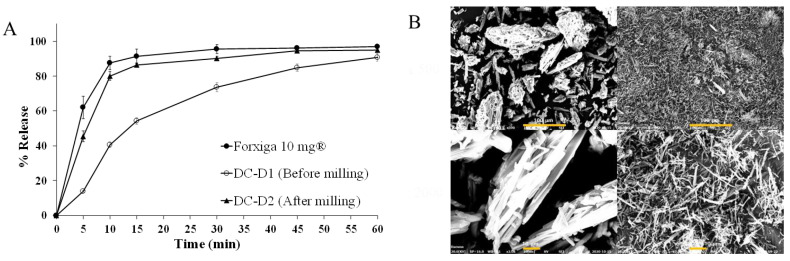
(**A**) Dissolution profiles of the DC-D1 tablet before and after milling of the dapagliflozin (DAP) cocrystal in pH 1.2. (**B**) SEM images of a DAP cocrystal before (left) and after (right) milling at two magnifications: 500 (top) and 2000 (bottom). Each value represents the mean ± standard deviation (*n* = 6).

To manufacture a DAP cocrystal tablet, DC tableting was reviewed at the initial stage. Diluents optimized for DC tableting were used for blending with the DAP cocrystal. Contrary to the rapid tablet disintegration, the initial dissolution of DC-D1 was slower compared to that of the reference tablet, Forxiga^®^ 10 mg, as shown in Figure 1. This phenomenon was attributed to the small DAP cocrystal surface area with a D90 diameter > 200 μm, as shown in the SEM image (Figure 1B). To improve the dissolution rate of DC-D1 with enhanced API surface area, the DAP cocrystal was pulverized with the jet mill, resulting in a reduced D90 diameter < 20 μm (Table S4) and an increased dissolution rate, similar to that of Forxiga^®^ 10 mg. However, this smaller size caused low compressibility and flowability, resulting in a high Carr’s index value (>30%) and the discontinuation of tablet manufacturing via the DC method. In addition, the tablet content uniformity was also not improved, regardless the excipient type or glidant increase, including DC-D2, D3, and D4 formation, showing large deviation values. Table S5 shows detailed information on manufacturability of DC formulations. To improve granule flowability and produce uniform tablet contents, the dry granulation method, namely roller compaction, was used.

### 3.5. Manufacturing DAP Cocrystal Tablets by Roller Compaction

#### 3.5.1. Effect of RC Manufacturing Parameters

RC is a powder agglomeration process used in a variety of industries, including pharmaceutical, mineral, and chemical [16]. In the pharmaceutical industry, RC is a suitable method for drug substances sensitive to water or other solvents, increasing the particle size to improve the granule flowability [17]. In the case of a roller system, conditions to synchronize the screw feeder with the speed of the roller must be achieved and applied to ensure a uniform and continuous flow, avoiding insufficient or excessive amounts of injected powder [18]. If there is no uniform and continuous flow or if the granule sheet from the roller system is not in a complete ribbon shape due to insufficient pressure, numerous fines may be included through the milling process, causing capping, chipping, lining, and mass deviation in the tableting process. However, if an excessive pressure is applied to the input powder, the dissolution may be delayed due to over-compressed granules [19]. Therefore, it is important to find the optimum conditions in which the feeding speed is synchronized, and the appropriate pressure is applied.

**Figure 2:**
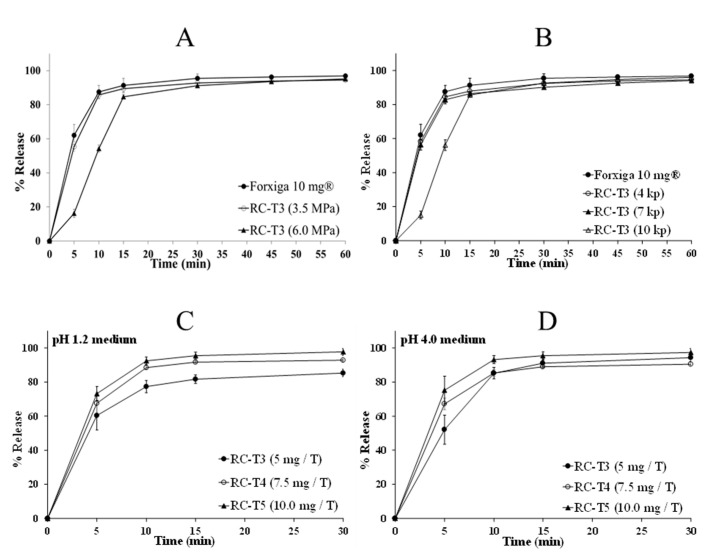
Effect of manufacturing parameters on the dapagliflozin (DAP) dissolution profiles. (**A**) Roller pressures (3.5 Mpa and 6.0 Mpa) with RC-T3 formulation with pH 1.2. (**B**) Tablet hardness (4 kp, 7 kp, and 10 kp) with RC-T3 formulation in pH 1.2, and Crospovidone contents (RC-T3, T4, T5) in (**C**) pH 1.2 and (**D**) pH 4.0. The experiment was conducted in USP Apparatus 1 at 50 rpm and in 900 mL dissolution media at 37.0 °C. Each value represents the mean ± standard deviation (*n* = 6).

The study searching for the optimal manufacturing parameters of RC was conducted in terms of roll rotation speed, screw rotation speed, and roller pressure with RC-T3 formulation. When the mixture was directly inserted between the rolls running at rotation speeds of 2, 5, and 8 rpm, 5 rpm was the most appropriate speed, whereas 2 and 8 rpm induced delayed production time and roller contamination (Table S6). The roll rotation speed was therefore fixed at 5 rpm, and the rotation speed of the screw feeder was evaluated at 1, 4, 7, and 10 rpm. In the case of 1 and 4 rpm, the sheet manufacturing was not identical; it was broken and unevenly generated, showing that these rotation speeds were not sufficient to inject the mixture into rolls. In contrast, at 10 rpm, the mixture was not loaded in the screw feeder, resulting in the mixture clustering along the inner wall of the chamber due to the high-speed rotation. Hence, the appropriate rotation of the screw feeder was 7 rpm, sufficient to achieve synchronization with a roll rotation of 5 rpm [20]. The roller pressure effect was evaluated by feeding the same mixture at 1.0, 3.5, and 6.0 MPa; 1.0 MPa was not sufficient for the formation of a ribbon-shaped sheet due to the low pressure applied to the mixture. In contrast, a smooth manufacturing process was possible at 3.5 MPa and 6.0 MPa, achieving a uniform ribbon sheet product. The applied roller pressure effect was evaluated using a dissolution test, as shown in Figure 2A. A significant delay in DAP release was found for the tablet at 6.0 MPa, when compared to that at 3.5 MPa. The delayed dissolution profile at 6.0 MPa stems from over-compressed granules, preventing the release of DAP cocrystals into the media [21]. Therefore, it was determined that a roller pressure of 3.5 MPa, roll rotation speed of 5 rpm, and screw feeder rotation speed of 7 rpm were most suitable for the RC process. Flowability and contents uniformity test of RC formulation are given in Table S7. The granule flowability was improved to a greater extent in RC-T3, in comparison to not only DC formulations but also RC-T1 and RC-T2, by excluding mannitol, which is not suitable for the RC process.

#### 3.5.2. Effect of Tablet Hardness

Tablet hardness of 1 and 13 kp was not appropriate due to friability and laminating problems. The dissolution results of tablet hardness of 4, 7, and 10 kp are shown in Figure 2B. Tablets of 4 and 7 kp hardness showed similar DAP release results as Forxiga^®^ 10 mg, whereas the dissolution of 10 kp tablets was delayed by 10 min. It is known that excipients for the dry granulation process are usually dependent on external pressure, meaning the pressure applied must be within the proper range. Lactose anhydrous and microcrystalline cellulose, used as diluents, were all excipients for dry granulation. The granule’s density in the manufactured ribbon sheet was determined according to the roller pressure applied during the RC process, which was confirmed through the dissolution test. In addition, the disintegration time of the uncoated tablet markedly varied depending on the tableting pressure. Therefore, it was confirmed that the pressure applied twice to the granules through both the RC and tableting process was stored in the diluents, whose microenvironment properties were elastically modified under external pressure.

#### 3.5.3. In Vitro Dissolution Test of DAP Cocrystal and Forxiga^®^ 10 mg Tablets

**Figure 3:**
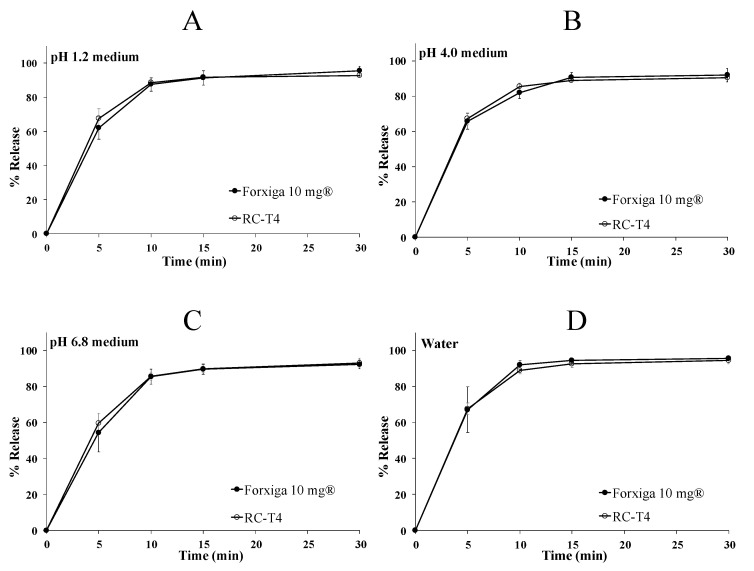
Dapagliflozin (DAP) dissolution profiles from Forxiga^®^ 10 mg and RC-T4 in pH 1.2 (**A**), pH 4.0 (**B**), pH 6.8 (**C**), and distilled water (**D**). The experiment was conducted in USP Apparatus 1 at 50 rpm and in dissolution media at 37.0 °C. Each value represents the mean ± standard deviation (*n* = 6).

Based on the fixed manufacturing process and RC-T4 formulation, research showed that the amount of crospovidone was 5 mg, 7.5 mg, and 10 mg for T3, T4, and T5, respectively. Figure 2C,D show that the crospovidone increase proportionally influenced the initial dissolution rate of the tablet. In particular, RC-T4 showed the most similar initial dissolution pattern to that of Forxiga^®^ 10 mg in the pH 1.2 medium. To predict the release pattern inside the human GI tract, the dissolution test was performed at pH 1.2, 4.0, 6.8, and water medium with regard to Forxiga^®^ 10 mg and RC-T4. Figure 3 shows that after 15 min, more than 85% DAP was released from all dissolution test media, and dissolution rate differences of Forxiga^®^ 10 mg and RC-T4 were within 5% at all time points. Due to the DAP cocrystal and DAP-PH solubility, which is not affected by pH, the rapid DAP release pattern was observed in all dissolution media.

### 3.6. Stability Assessment

#### 3.6.1. Stability Assessment of DAP Cocrystal Tablets

Accelerated and long-term stability tests were carried out for RC-T4. The DAP content did not change in both accelerated and long-term stability tests, ranging from approximately 98.3% to 100.2%. Total impurities were <0.2% in both test conditions, representing stability acceptance.

#### 3.6.2. Stability Assessment of Cocrystal Structure Maintenance

**Figure 4:**
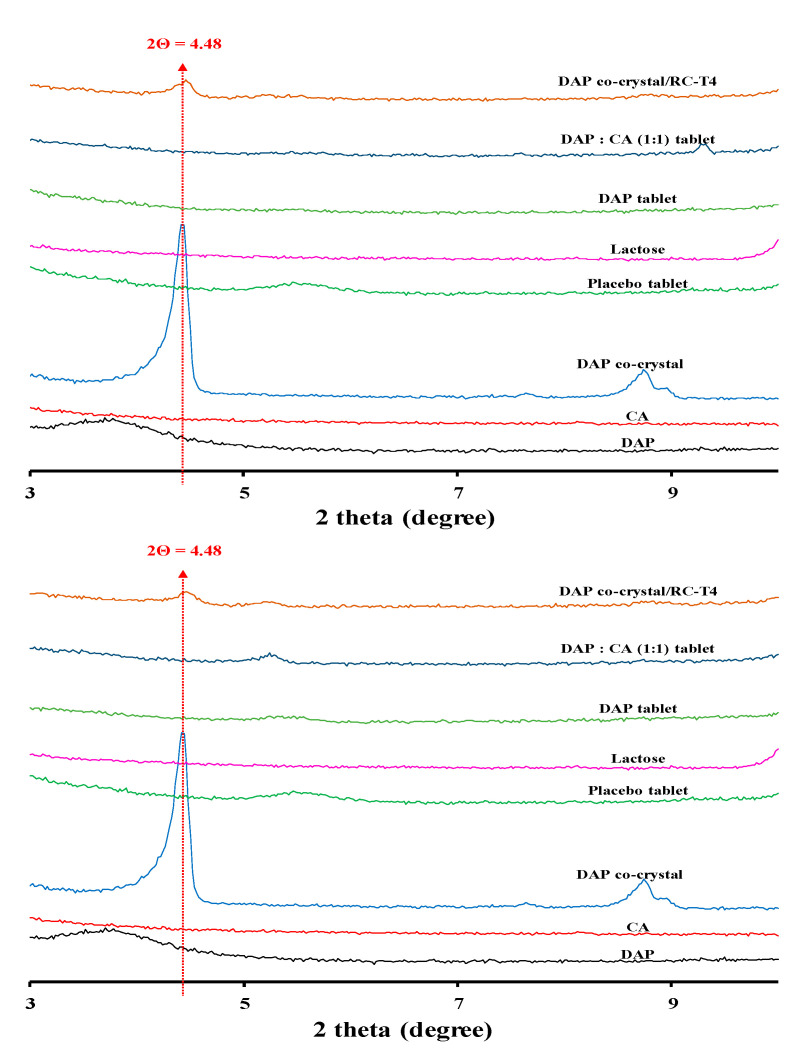
X-ray diffraction (XRD) patterns of RC-T4 with various groups under the long-term (top, 6 months) and accelerated condition (bottom, 6 months).

XRD analysis for identifying the cocrystal structure preservation in the tablet is shown in Figure 4. The results showed that a peak of 4.48°, the characteristic signal of the cocrystal structure, was observed in RC-T4 after accelerated and long-term storage conditions. The peak intensity of 4.48° was maintained for 6 months during the stability test period, indicating that the DAP cocrystal structure did not change, and hydrogen bonding remained in spite of the surrounding excipients.

### 3.7. In Vivo Comparative PK Studies in Beagle Dogs and Healthy Human Volunteers

#### 3.7.1. PK Study in Beagle Dogs

**Figure 5:**
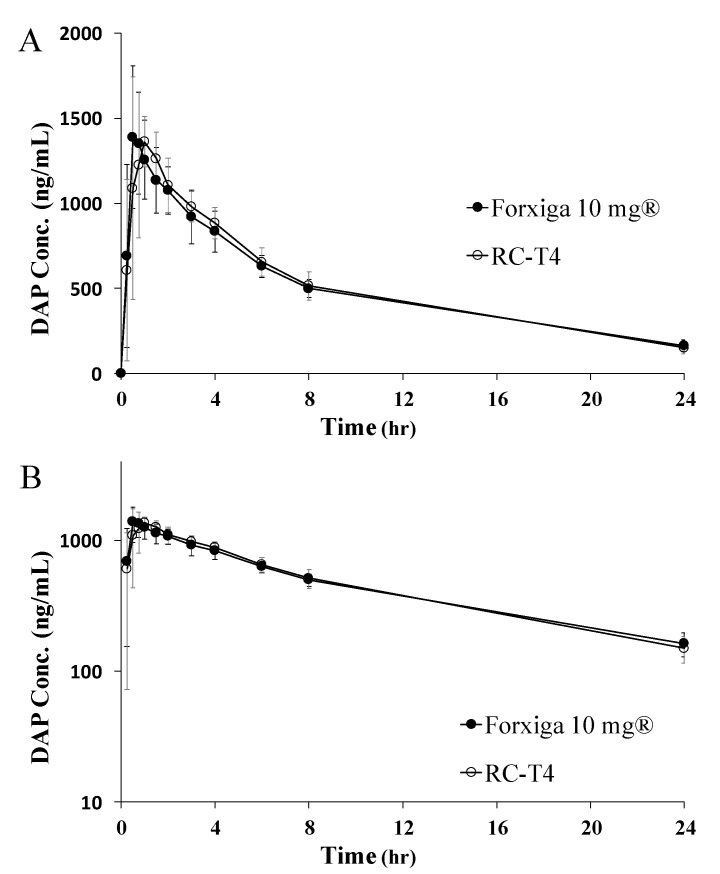
Average plasma concentration vs. time profiles of DAP after oral administration of Forxiga^®^ 10 mg (●) and RC-T4 (○) in beagle dogs ((**A**): linear scale, (**B**): semilog scale). Values are presented as the mean ± standard deviation (*n* = 12).

To investigate the formulation effects on the PK profile, the selected tablets, RC-T4 and Forxiga^®^ 10 mg, were administered orally as a test and reference formulations, respectively, to 12 beagle dogs in a crossover design. The PK parameters of DAP after oral administration of Forxiga^®^ 10 mg and RC-T4 in beagle dog and the plasma concentration curve over time are shown in Table 4 and Figure 5, respectively. The AUC values indicate that the reference and test formulations show similar bioavailability of 11,919 ± 1469 and 12,116 ± 1550 ng·h/mL, respectively. The C_max_ values were 1500 ± 308 and 1504 ± 233 ng/mL for the reference and test formulations, respectively. The T_max_ results indicate that both formulations reached the highest DAP blood concentration within 1 h, consistent with previously reported values [22,23]. Bioequivalence was further evaluated by statistically comparing the relative AUC ratio and 90% confidence intervals (CI) for logarithmic-transformed data. The AUC test/reference (T/R) ratio, defined as the relative ratio of the corresponding RC-T4 value, divided by the equivalent Forxiga^®^ 10 mg value and the 90% CI, were 1.017 and 0.9899–1.045, respectively. The C_max_ T/R ratio and 90% CI were 1.019 and 0.9301–1.115, respectively. Based on these statistical analysis, two formulations of Forxiga^®^ 10 mg and RC-T4 showed similar bioavailability, showing the bioequivalence. Therefore, the RC-T4 was selected as the final test formulation for a human PK study [24].

#### 3.7.2. PK Study in Healthy Human Volunteers

**Figure 6:**
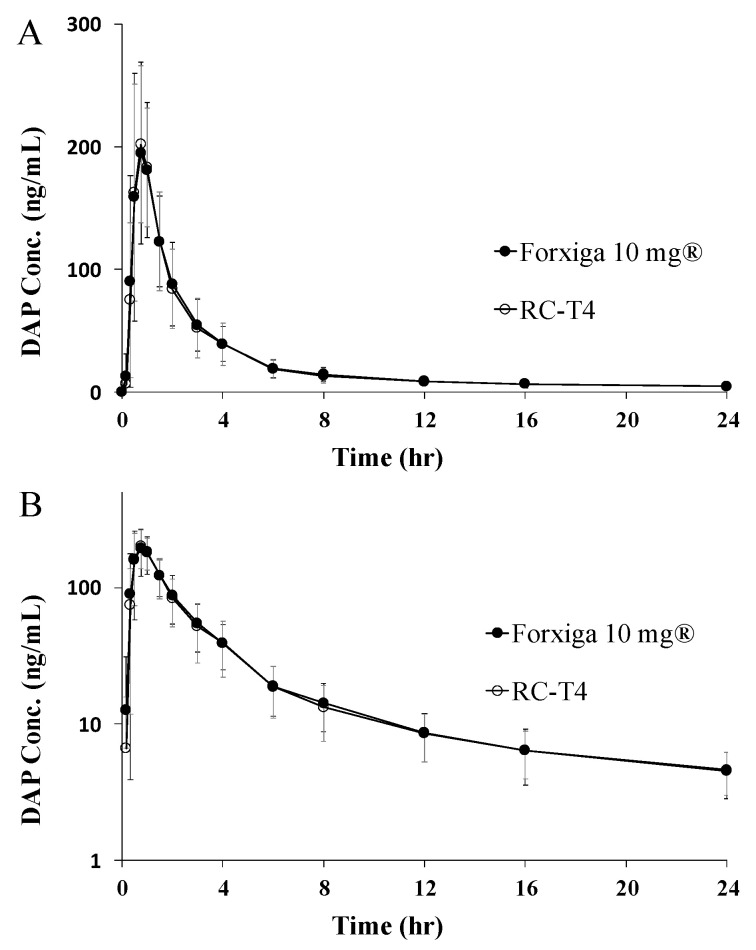
Average plasma concentration vs. time profiles of dapagliflozin (DAP) after oral administration of Forxiga 10 mg (●) and RC-T4 (○) in healthy volunteers ((**A**): linear scale, (**B**): semilog scale). Each value represents the mean ± standard error (*n* = 37).

RC-T4 and Forxiga^®^ 10 mg, validated in beagle dogs, were also administered orally as a test and reference tablet to 37 healthy adult male volunteers, respectively. The PK parameters of DAP after oral administration of Forxiga^®^ 10 mg and RC-T4 and the plasma concentration curves in healthy volunteers are shown in Table 5 and Figure 6, respectively. The AUC values were 592.01 ± 165.56 and 618.74 ± 184.71 ng·h/mL for Forxiga^®^ 10 mg and RC-T4, respectively. In addition, the C_max_ values indicate that the reference and test formulations have similar blood concentrations, 223.44 ± 54.75 and 229.41 ± 69.13 ng/mL, respectively. The bioequivalence of RC-T4 and Forxiga^®^ 10 mg in humans was further evaluated by statistically comparing the relative ratio of the AUC and 90% CI for logarithmic-transformed data. The T/R ratio for AUC and the 90% CI was 1.041 and 1.012–1.071, respectively, whereas that for C_max_ and 90% CI was 1.013 and 0.911–1.127, respectively. According to FDA guidelines, the 90% CI of the log-transformed average difference was between 0.80 and 1.25, so the two formulations were judged to be equivalent. Bioequivalence between Forxiga^®^ 10 mg and RC-T4, was simultaneously confirmed through in vivo tests in beagle dogs and human volunteers. Interestingly, the blood concentration in human volunteers was much lower as compared with beagle dogs. According to the Biopharmaceutical Classification System (BCS), DAP belongs to Class III with high solubility and low permeability. The PK profile of DAP co crystal and DAP-PH are expected to be more affected by dissolution rate than absorption factor. It appeared that both DAP and DAP-PH were rapidly dissolved in DAP and salts in the stomach, leading to rapid absorption in the gastrointestinal tract. The Forxiga^®^ 10 mg and RC-T4 showed similar bioavailability and C_max_ within 1 h. In Vitro dissolution test performed in 4 different pH media showed that the DAP cocrystal and DAP-PH had little effect on dissolution. However, in the in vivo studies, where the PK pattern of beagle dogs, which usually have higher gastric pH (2.7 to 8.3), was similar to humans in terms of T/R ratio, who have a relatively low gastric pH of 1–2 [25,26]. In addition, any unwanted side effects were not observed during human PK studies.

## 4. Conclusions

In this study, the hydrogen bonds in DAP cocrystal structure was confirmed through XRD and FTIR. The physical properties of the cocrystal against heat and moisture were superior to those of DAP and DAP-PH. To improve granule flowability and achieve a continuous manufacturing system, optimization of the RC parameters was tested and established. Moreover, it was confirmed that the cocrystal structure was maintained during the entire stability test for the final selected formulation, RC-T4. Similar Forxiga^®^ 10 mg and RC-T4 PK patterns were identified in beagle dogs. Comparative PK studies of RC-T4 and reference tablets using crossover design in human volunteers showed bioequivalence with regard to the extent of bioavailability and C_max_.

## Figures and Tables

**Table 1:** Formulation compositions (mg) of the DAP cocrytal-loaded tablet via DC process.

Ingredients	Function	DC-D1	DC-D2	DC-D3	DC-D4	DC-D5
DAP cocrystal (before milling)	API	14.7	-	-	-	-
DAP cocrystal (after milling)	API	-	14.7	14.7	14.7	14.7
Microcrystalline cellulose	Filler	141.3	141.3	141.3	76.3	141.3
Lactose anhydrous	Filler	-	-	65.0	65.0	-
Mannitol	Filler	65.0	65.0	-	65.0	65.0
Crospovidone	Disintegrant	5.0	5.0	5.0	5.0	5.0
Colloidal silicon dioxide	Lubricant	4.0	4.0	4.0	10.0	10.0
Magnesium stearate	Lubricant	5.0	5.0	5.0	5.0	5.0
Opadry 03B62323	Coating agent	10.0	10.0	10.0	10.0	10.0

**Table 2:** Formulation compositions (mg) of the DAP cocrystal-loaded tablet via RC process.

Ingredients	Function	RC-T1	RC-T2	RC-T3	RC-T4	RC-T5
DAP cocrystal	API	14.7	14.7	14.7	14.7	14.7
Microcrystalline cellulose	Filler	142.5	76.3	150.0	147.5	145
Lactose anhydrous	Filler	-	65.0	61.3	61.3	61.3
Mannitol	Filler	63.8	65.0	-	-	-
Crospovidone	Disintegrant	5.0	5.0	5.0	7.5	10.0
Colloidal silicon dioxide	Lubricant	4.0	4.0	4.0	4.0	4.0
Magnesium stearate	Lubricant	5.0	5.0	5.0	5.0	5.0
Opadry 03B62323	Coating agent	10.0	10.0	10.0	10.0	10.0

**Table 3:** Equilibrium solubility and dose number of dapagliflozin (DAP), dapagliflozin propanediol monohydrate (DAP-PH), and DAP cocrystal in various aqueous media.

Active Ingredients	Media	Equilibrium Solubility (C_S_, mg/mL)	D_0_ *
DAP	pH 1.2	0.90	0.04
	pH 4.0	0.94	0.04
	pH 6.8	0.81	0.05
	Water	0.86	0.05
DAP-PH	pH 1.2	1.68	0.02
	pH 4.0	1.74	0.02
	pH 6.8	1.60	0.03
	Water	1.70	0.02
DAP cocrystal	pH 1.2	2.20	0.02
	pH 4.0	2.62	0.02
	pH 6.8	1.73	0.02
	Water	2.33	0.02

* Calculated by Equation (1).

**Table 4:** PK parameters of dapagliflozin (DAP) after oral administration of Forxiga^®^ 10 mg and RC-T4 in beagle dogs.

Parameter	Forxiga^®^ 10 mg	RC-T4	T/R ratio ^f^	CV-ANOVA ^g^
AUC_0–24_ (ng·h/mL) ^a^	11919 ± 1469	12116 ± 1550	1.017 (0.9899~1.045)	4.044
AUC_0–∞_ (ng·h/mL) ^b^	13617 ± 2013	13364 ± 1965		
C_max_ (ng/mL) ^c^	1500 ± 308	1504 ± 233	1.019 (0.9310~1.115)	11.729
t_max_ (h) ^d^	0.50 ± 0.75	0.75 ± 0.50		
t_1/2_ (h) ^e^	9.47 ± 1.40	8.50 ± 0.72		

Each value represents the mean ± S.D. (*n* = 12). ^a^ Area under the plasma concentration-time curve at 0–24 h. ^b^ area under the plasma concentration-time curve at 0–∞ h. ^c^ measured maximum plasma concentration at 0–24 h. ^d^ time to reach peak concentration at first dosing interval. ^e^ half-life. ^f^ calculated from mean values and 90% confidence interval (CI) values of logarithmic transformed data of reference (Forxiga^®^ 10 mg) and test (DAP cocrystal/RC-T4). ^g^ The coefficient of variation or relative standard deviation is the standard deviation expressed as a percentage of the mean.

**Table 5:** PK parameters of dapagliflozin (DAP) after oral administration of Forxiga^®^ 10 mg and RC-T4 in healthy volunteers.

Parameter	Forxiga^®^ 10 mg	RC-T4	T/R Ratio ^f^	CV-ANOVA ^g^
AUC_0–24_ (ng·h/mL) ^a^	592.01 ± 165.56	618.74 ± 184.71	1.0413 (1.0121~1.0713)	6.425
AUC_0–∞_ (ng·h/mL) ^b^	626.21 ± 160.53	651.65 ± 183.18		
C_max_ (ng/mL) ^c^	223.44 ± 54.75	229.41 ± 69.13	1.0135 (0.9113~1.1270)	27.032
t_max_ (h) ^d^	0.75 ± 0.75	0.75 ± 0.75		
t_1/2_ (h) ^e^	12.64 ± 4.47	11.27 ± 3.14		

Each value represents the mean ± standard deviation (*n* = 37). ^a^ Area under the plasma concentration-time curve at 0–24 h. ^b^ area under the plasma concentration-time curve at 0–∞ h. ^c^ measured maximum plasma concentration at 0–24 h. ^d^ time to reach peak concentration at first dosing interval. ^e^ half-life. ^f^ calculated from mean values and 90% confidence interval (CI) values of logarithmic transformed data of reference (Forxiga^®^ 10 mg) and test (DAP cocrystal/RC-T4). ^g^ The coefficient of variation or relative standard deviation is the standard deviation expressed as a percentage of the mean.

## Data Availability

The data presented in this study are available upon request.

## Supplementary Materials

The following are available online at https://www.mdpi.com/1999-4923/13/1/70/s1. Figure S1. Overlay XRD results of citric acid (black), zinc chloride (green), L-tryptophan (purple), sodium lauryl sulfate (blue), and L-proline (red), Figure S2: (a) XRD patterns of DAP (black), CA (red), DAP cocrystal (blue), and DAP:CA (1:1) physical mixture (green). (b) FTIR spectra of DAP (black), DAP-PH (red), DAP cocrystal (blue), and CA (green). (c) DSC thermograms of DAP (black), DAP-PH (red), and DAP cocrystal (blue). (d) TGA thermograms of DAP (black), DAP-PH (red), and DAP cocrystal (blue), Figure S3: Water sorption isotherms for DAP (blank), DAP-PH (red) and DAP cocrystal (blue), Figure S4: Appearance test of DAP, DAP-PH, and DAP cocrystal under three conditions: (1) 60 °C, closed (2) 25 °C, 90% RH, open (3) 40 °C, 75% RH, open; after 1 week, Table S1. Coformers used in the cocrystal screen with DAP, Table S2. Composition for various aqueous media, Table S3. DAP content and impurity of DAP, DAP-PH, and DAP cocrystal under three conditions: (1) 60 °C, closed (2) 25 °C, 90% RH, open (3) 40 °C, 75% RH, open; after 4 weeks, Table S4. Particle size distribution of DAP cocrystal before and after milling process, Table S5. Detailed information on manufacturability of DC formulations, Table S6. Manufacturing parameters screening for roller compaction process, Table S7. Flowability and contents uniformity test of roller compaction (RC) formulation.

## Abbreviations

API	Active pharmaceutical ingredient
CA	Citric acid
DAP	Dapagliflozin
DAP-PH	Dapagliflozin propanediol monohydrate
DC	Direct compression
RC	Roller compaction
XRD	X-ray diffraction
FTIR	Fourier transform infrared spectroscopy
DSC	Differential scanning calorimetry
TGA	Thermogravimetric analysis
DVS	Dynamic vapor sorption
SGLTs	Sodium glucose transport proteins
SEM	Scanning electron microscopy
RH	Relative humidity
PK	Pharmacokinetics
AUC	Area under the plasma concentration-time curve
C_max_	Maximum plasma concentration
T_max_	Time to reach peak concentration
T/R ratio	Test/reference ratio

## References

[1] Shin S.J., Chung S., Kim S.J., Lee E.M., Yoo Y.H., Kim J.W., Ahn Y.B., Kim E.S., Moon S.D., Kim M.J. (2016). Effect of Sodium-Glucose Co-Transporter 2 Inhibitor, Dapagliflozin, on Renal Renin-Angiotensin System in an Animal Model of Type 2 Diabetes. PLoS ONE.

[2] Jabbour S., Goldstein B. (2008). Sodium glucose co-transporter 2 inhibitors: Blocking renal tubular reabsorption of glucose to improve glycaemic control in patients with diabetes. Int. J. Clin. Pract..

[3] Singh D., Tiwary A.K., Bedi N. (2019). Canagliflozin loaded SMEDDS: Formulation optimization for improved solubility, permeability and pharmacokinetic performance. J. Pharm. Investig..

[4] Kasichayanula S., Chang M., Hasegawa M., Liu X., Yamahira N., LaCreta F., Imai Y., Boulton D. (2011). Pharmacokinetics and pharmacodynamics of dapagliflozin, a novel selective inhibitor of sodium-glucose co-transporter type 2, in Japanese subjects without and with type 2 diabetes mellitus. Diabetesobes. Metab..

[5] Filippatos T.D., Liberopoulos E.N., Elisaf M.S. (2015). Dapagliflozin in patients with type 2 diabetes mellitus. Ther. Adv. Endocrinol. Metab..

[6] El-Bagory I., Alruwaili N.K., Elkomy M.H., Ahmad J., Afzal M., Ahmad N., Elmowafy M., Alharbi K.S., Alam M.S. (2019). Development of novel dapagliflozin loaded solid self-nanoemulsifying oral delivery system: Physiochemical characterization and in vivo antidiabetic activity. J. Drug Deliv. Sci. Technol..

[7] Deng J.-H., Lu T.-B., Sun C.C., Chen J.-M. (2017). Dapagliflozin-citric acid cocrystal showing better solid state properties than dapagliflozin. Eur. J. Pharm. Sci..

[8] Domingos S., André V., Quaresma S., Martins I.C., Minas da Piedade M.F., Duarte M.T. (2015). New forms of old drugs: Improving without changing. J. Pharm. Pharmacol..

[9] Matsumoto K., Hasegawa T., Ohara K., Takei C., Akimoto M. (2020). Roles of CYP2C9 and its variants (CYP2C9* 2 and CYP2C9* 3) in the metabolism of 6-methoxy-2-napthylacetic acid, an active metabolite of the prodrug nabumetone. J. Pharm. Investig..

[10] Kim P., Kim G.-Y., Cho M.-Y., Lee M.-J., Choi G.J. (2020). Manufacture and characterization of two distinct quasi-polymorphs of empagliflozin. J. Cryst. Growth.

[11] Saha S., Desiraju G.R. (2018). Acid···Amide supramolecular synthon in cocrystals: From spectroscopic detection to property engineering. J. Am. Chem. Soc..

[12] Tothadi S., Desiraju G.R. (2012). Synthon modularity in 4-hydroxybenzamide–dicarboxylic acid cocrystals. Cryst. Growth Des..

[13] Aitipamula S., Wong A.B., Chow P.S., Tan R.B. (2014). Cocrystallization with flufenamic acid: Comparison of physicochemical properties of two pharmaceutical cocrystals. CrystEngComm.

[14] Yadav A., Shete A., Dabke A., Kulkarni P., Sakhare S. (2009). Co-crystals: A novel approach to modify physicochemical properties of active pharmaceutical ingredients. Indian J. Pharm. Sci..

[15] Diniz L.F., Souza M.S., Carvalho P.S., da Silva C.C., D’Vries R.F., Ellena J. (2018). Novel Isoniazid cocrystals with aromatic carboxylic acids: Crystal engineering, spectroscopy and thermochemical investigations. J. Mol. Struct..

[16] Mitchell S.A., Reynolds T.D., Dasbach T.P. (2003). A compaction process to enhance dissolution of poorly water-soluble drugs using hydroxypropyl methylcellulose. Int. J. Pharm..

[17] Kleinebudde P. (2004). Roll compaction/dry granulation: Pharmaceutical applications. Eur. J. Pharm. Biopharm..

[18] Guigon P., Simon O. (2003). Roll press design—Influence of force feed systems on compaction. Powder Technol..

[19] Park M.S., Choi D.H. (2020). Application of mechanism-based modeling to predict drug quality during the pharmaceutical unit operations of granulation and compression: A review. J. Pharm. Investig..

[20] Pishnamazi M., Casilagan S., Clancy C., Shirazian S., Iqbal J., Egan D., Edlin C., Croker D.M., Walker G.M., Collins M.N. (2019). Microcrystalline cellulose, lactose and lignin blends: Process mapping of dry granulation via roll compaction. Powder Technol..

[21] Rambali B., Baert L., Jans E., Massart D. (2001). Influence of the roll compactor parameter settings and the compression pressure on the buccal bio-adhesive tablet properties. Int. J. Pharm..

[22] Kasichayanula S., Liu X., LaCreta F., Griffen S.C., Boulton D.W. (2014). Clinical pharmacokinetics and pharmacodynamics of dapagliflozin, a selective inhibitor of sodium-glucose co-transporter type 2. Clin. Pharmacokinet..

[23] Jang J.-H., Jeong S.-H., Cho H.-Y., Lee Y.-B. (2019). Comparison of UPLC-MS/MS and HPLC-UV methods for the determination of zaltoprofen in human plasma. J. Pharm. Investig..

[24] Jeong S.-H., Jang J.-H., Cho H.-Y., Oh I.-J., Lee Y.-B. (2020). A sensitive UPLC–ESI–MS/MS method for the quantification of cinnamic acid in vivo and In Vitro: Application to pharmacokinetic and protein binding study in human plasma. J. Pharm. Investig..

[25] Akimoto M., Nagahata N., Furuya A., Fukushima K., Higuchi S., Suwa T. (2000). Gastric pH profiles of beagle dogs and their use as an alternative to human testing. Eur. J. Pharm. Biopharm..

[26] Kosugi Y., Yamamoto S., Sano N., Furuta A., Igari T., Fujioka Y., Amano N. (2015). Evaluation of acid tolerance of drugs using rats and dogs controlled for gastric acid secretion. J. Pharm. Sci..

