# Supplementary material for: Improved Manufacturability and In Vivo Comparative Pharmacokinetics of Dapagliflozin Cocrystals in Beagle Dogs and Human Volunteers
Source: Pharmaceutics. 2021 Jan 7;13(1):70. doi: 10.3390/pharmaceutics13010070 (PMC7825777; doi:10.3390/pharmaceutics13010070)
Supplement: Supplementary file 1 [file pharmaceutics-13-00070-s001.pdf]

pharmaceutics

MDPI

Supplement

Improved Manufacturability and In Vivo Comparative Pharmacokinetics of Dapagliflozin Co-Crystals in Beagle Dogs and Human Volunteers

Sangho Cho Jeongwook Lee, Yongwon Yoo, Minyong Cho, Seil Sohn and Beom-Jin Lee

Table S1. Coformers used in the co-crystal screen with DAP.

**Adenine.**	**Gentisic acid**	**D,L-mandelic acid**	**Sorbic acid**
Adipic acid	L-glutamic acid	D-mannitol	Sorbitol
4-aminobenzoic acid	D-glucose	Monosodium glutamate	Succinic acid
Ammonium chloride	Glutamine	Mucic (galacteric) acid	Sucralose
Arginine	Glutaric acid	Nicotinamide	Sucrose
L-ascorbic acid	Glycine	Oxalic acid	L-tartaric acid
Asparagine	Glycolic acid	L-proline	Thiamine hydrochloride
Aspartame	Hippuric acid	Propyl gallate	L-threonine
Benzoic acid	Histidine	L-pyroglutamic acid	Tromethamine hydrochloride
Betaine hydrochloride	1-hydroxy-2-naphthoic acid	Riboflavin	L-tryptophan
(+)-camphoric acid	L-lactic acid	Saccharin	Urea
Capric acid	L-leucine	Salicylic acid	L-valine
Choline chloride	L-lysine	Sebacic acid	Vanillin
Cinnamic acid	Maleic acid	L-serine	Xylitol
Citric acid	L-malic acid	Sodium chloride	Zinc chloride
Cyclamic acid	Malonic acid	Sodium lauryl sulfate	

Table S2. Composition for dissolution media.

**Composition**	**pH 1.2**	**pH 4.0**	**pH 6.8**
Sodium chloride (mM)	34.2	-	-
Hydrochloric acid conc. (mM)	82.4	-	-
Sodium acetate trihydrate (mM)	-	9.0	-
Acetic acid glacial (mM)	-	41.0	-
Potassium dihydrogen phosphate (mM)	-	-	50.0
Sodium hydroxide (mM)	-	-	23.5

Table S3. Results of DAP content and impurity of DAP, DAP-PH and DAP co-crystal in 3 conditions; 1) 60 °C, closed 2) 25 °C, 90 % RH, open 3) 40 °C, 75 % RH, open; after 4 week.

**Conditions**	**API Type**	**Time Points (week)**	**DAP Contents (%)**	**Contents of Total DAP Impurities (%) ***
60 °C, closed	DAP	0	99.4 ± 1.0	0.1 ± 0.1
		4	98.1 ± 1.0	0.2 ± 0.1
	DAP-PH	0	99.6 ± 1.0	0.1 ± 0.1
		4	99.4 ± 1.1	0.1 ± 0.1

	DAP co-crystal	0	99.3 ± 1.0	0.1 ± 0.1
		4	99.1 ± 1.1	0.1 ± 0.1
25 °C, 90 % RH	DAP	0	99.5 ± 1.0	0.1 ± 0.1
		4	99.1 ± 1.0	0.1 ± 0.1
	DAP-PH	0	99.4 ± 1.0	0.1 ± 0.1
		4	99.6 ± 1.2	0.1 ± 0.1
	DAP co-crystal	0	99.7 ± 1.0	0.1 ± 0.1
		4	99.9 ± 1.2	0.1 ± 0.1
40 °C, 75 % RH	DAP	0	99.4 ± 0.8	0.1 ± 0.0
		4	99.0 ± 1.0	0.2 ± 0.1
	DAP-PH	0	99.6 ± 1.2	0.1 ± 0.1
		4	99.4 ± 1.1	0.1 ± 0.1
	DAP co-crystal	0	99.7 ± 1.3	0.1 ± 0.1
		4	99.6 ± 1.1	0.1 ± 0.1

* mean ± standard deviation (*n* = 3)

Table S4. Particle size distribution of DAP co-crystal before and after milling process.

.	D 10	D 50	D 90
Before milling (μm)	3.5 ± 1.6	83.4 ± 5.9	229.5 ± 11.9
After milling (μm)	1.3 ± 0.2	4.8 ± 0.6	15.5 ± 1.1

Table S5. Detailed information on manufacturability of DC formulations.

Characterization	DC-D1	DC-D2	DC-D3	DC-D4	DC-D5
Carr`s index (%)	33.2 ± 0.8	34.4 ± 1.0	34.6 ± 0.8	31.1 ± 1.0	30.5 ± 0.9
Contents uniformity (%)	12.3	11.0	10.7	11.0	10.4
Manufacturing troubleshooting	Mass deviation	Mass deviation	Mass deviation	Mass deviation	Mass deviation

Table S6. Manufacturing parameters screening for roller compaction process.

Parameter	Screen Range	Optimum Production Range
Roll rotation speed (RPM)	2.0~8.0	5.0
Screw feeder speed (RPM)	1.0~10.0	7.0
Roll pressure (MPa)	1.0~6.0	3.5~6.0

Table S7. Flowability and contents uniformity test of roller compaction (RC) formulation.

Characterization	RC-T1	RC-T2	RC-T3	RC-T4	RC-T5
Carr`s index (%)	27.2 ± 1.3	25.1 ± 1.1	18.0 ± 0.9	18.3 ± 0.7	19.1 ± 0.8
Contents uniformity (%)	6.4	5.4	3.3	3.2	3.4
Manufacturing trouble	Discontinuous manufacturing	-	-	-	-

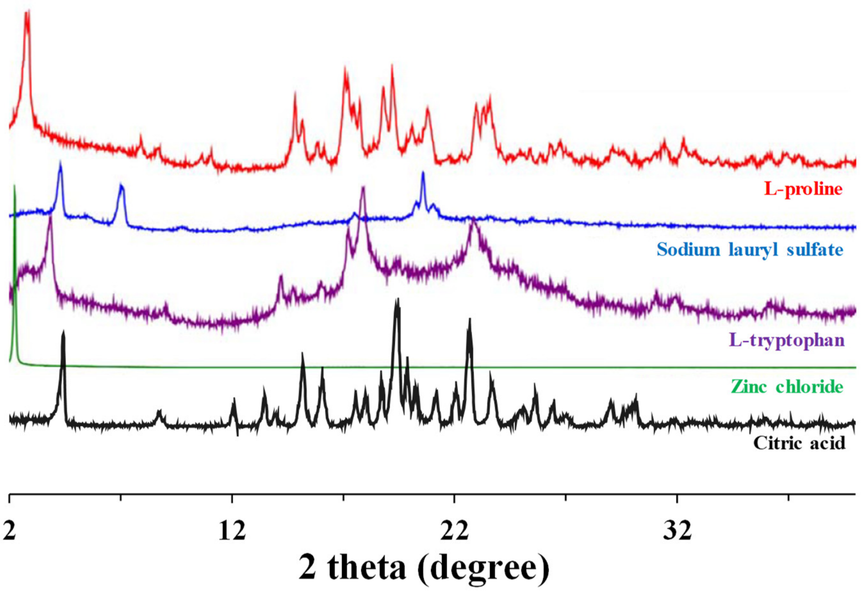

Figure S1. Overlay XRD results of citric acid(black), zinc chloride (green), L-tryptophan (purple), sodium lauryl sulfate (blue) and L-proline (red).

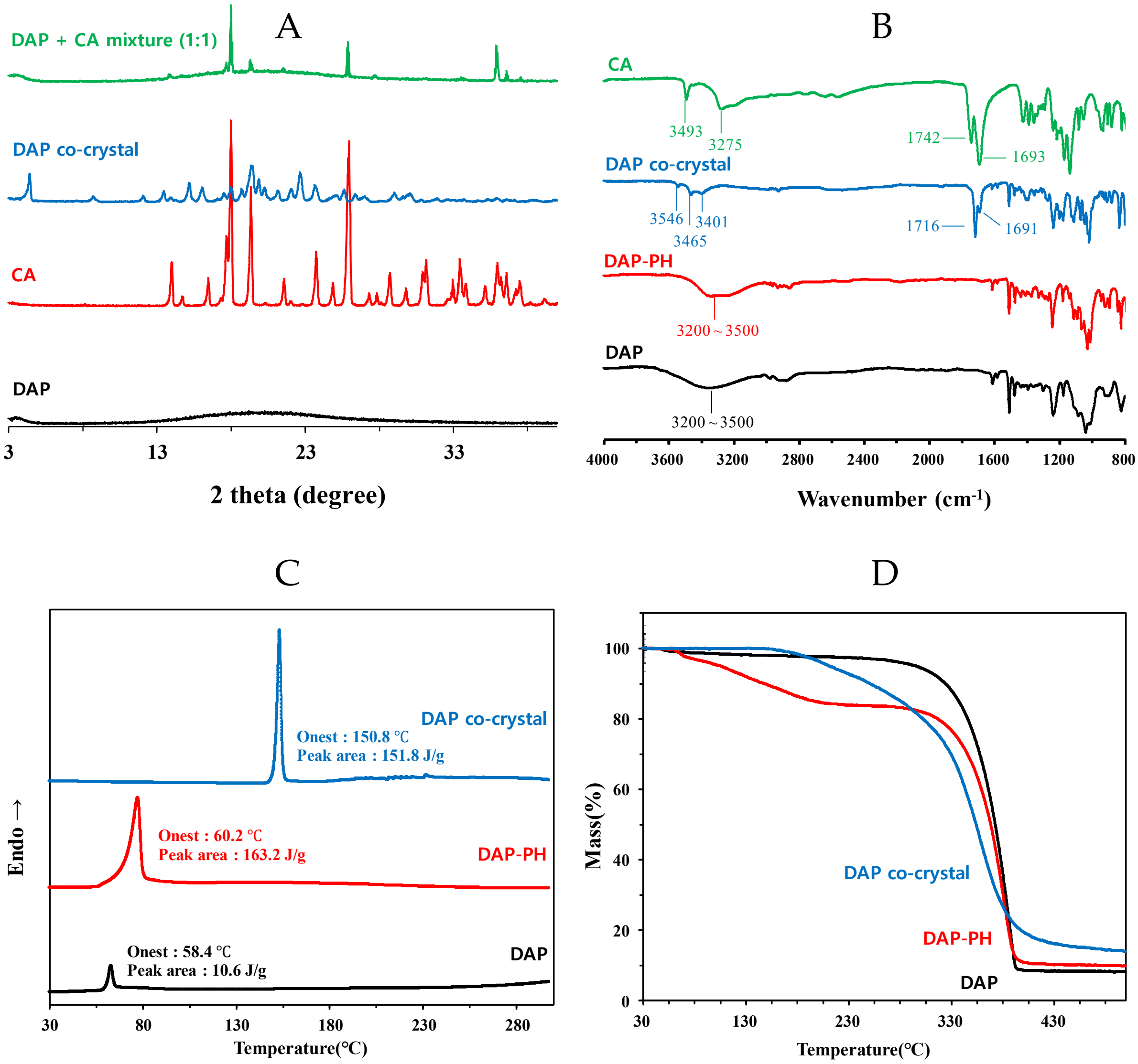

Figure S2. (**A**) XRD patterns of DAP (black), CA (red), DAP co-crystal (blue) and DAP: CA (1:1) physical mixture (green). (**B**) FTIR spectra of DAP (black), DAP-PH (red), DAP co-crystal (blue) and CA (green). (**C**) DSC thermograms of DAP (black), DAP-PH (red) and DAP co-crystal (blue). (**D**) TGA thermograms of DAP (black), DAP-PH (red) and DAP co-crystal (blue).

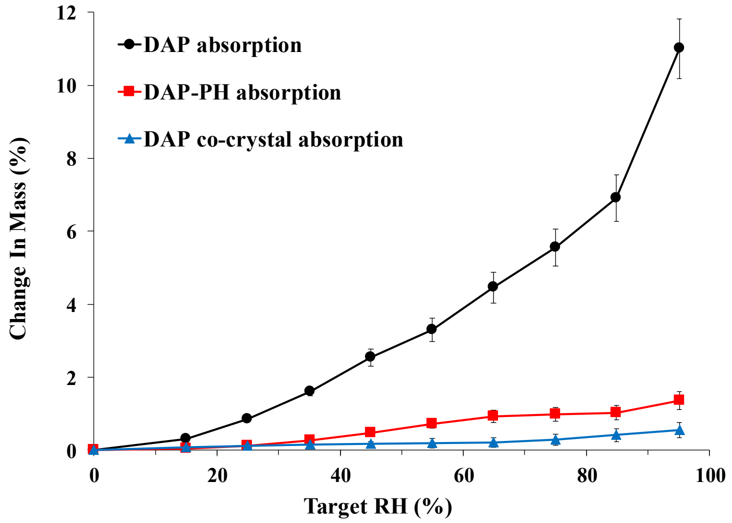

Figure S3. Water sorption isotherms for DAP (blank), DAP-PH (red) and DAP co-crystal (blue).

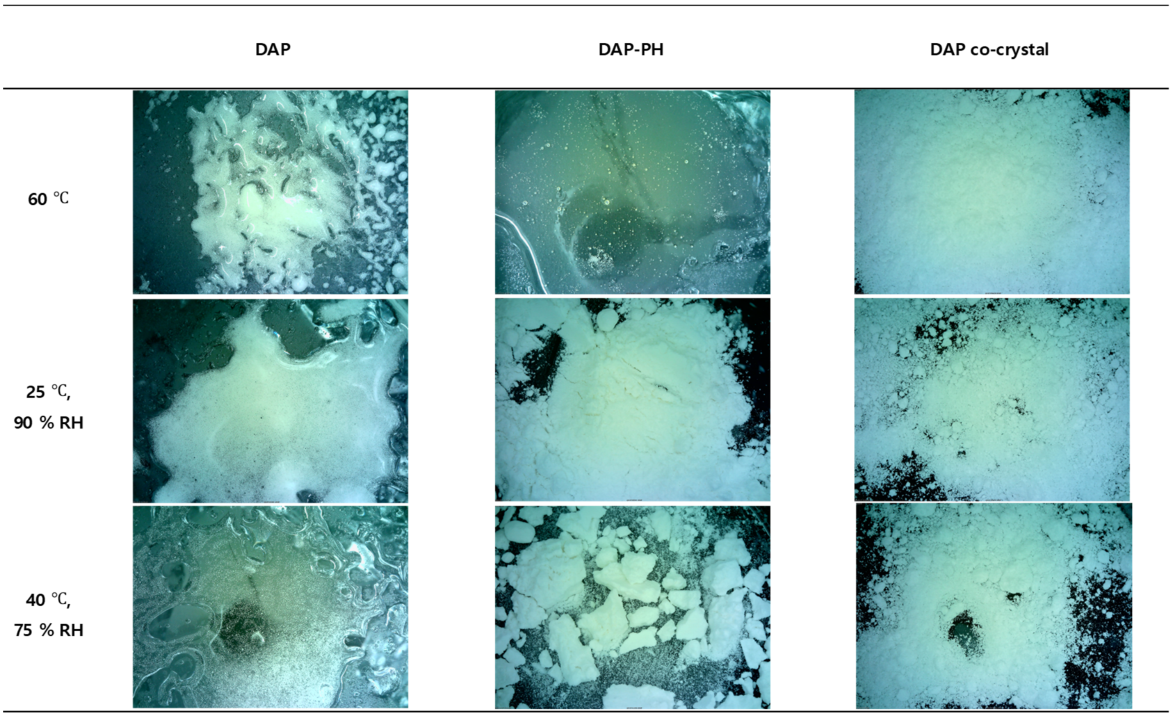

Figure S4. Appearance test of DAP, DAP-PH, and DAP co-crystal in 3 conditions; (1) 60 °C, closed (2) 25 °C, 90 % RH, open (3) 40 °C, 75 % RH, open; after 1 week.
